# Design and Manufacturing of a Disposable, Cyclo-Olefin Copolymer, Microfluidic Device for a Biosensor †

**DOI:** 10.3390/s19051178

**Published:** 2019-03-07

**Authors:** Jorge Prada, Christina Cordes, Carsten Harms, Walter Lang

**Affiliations:** 1 Institut für Mikrosensoren, -Aktoren und -Systeme, Universität Bremen, 28359 Bremen, Germany; wlang@imsas.uni-bremen.de; 2 Bremerhavener Institut für Angewandte Molekularbiologie, Hochschule Bremerhaven, 27568 Bremerhaven, Germany; christinacordes@hs-bremerhaven.de (C.C.); charms@hs-bremerhaven.de (C.H.)

**Keywords:** biosensors, Cyclo-Olefin Copolymer, cell heat-lysis, resistive temperature sensor, RNA hybridization, fluorescence detection

## Abstract

This contribution outlines the design and manufacturing of a microfluidic device implemented as a biosensor for retrieval and detection of bacteria RNA. The device is fully made of Cyclo-Olefin Copolymer (COC), which features low auto-fluorescence, biocompatibility and manufacturability by hot-embossing. The RNA retrieval was carried on after bacteria heat-lysis by an on-chip micro-heater, whose function was characterized at different working parameters. Carbon resistive temperature sensors were tested, characterized and printed on the biochip sealing film to monitor the heating process. Off-chip and on-chip processed RNA were hybridized with capture probes on the reaction chamber surface and identification was achieved by detection of fluorescence tags. The application of the mentioned techniques and materials proved to allow the development of low-cost, disposable albeit multi-functional microfluidic system, performing heating, temperature sensing and chemical reaction processes in the same device. By proving its effectiveness, this device contributes a reference to show the integration potential of fully thermoplastic devices in biosensor systems.

## 1. Introduction

The on-site, readily detection of hazardous bacteria by means of on-chip microfluidic systems has become a relevant trend in the development of Lab-on-Chip and Micro Analysis Devices [1]. Their reduced analysis time and cost in comparison to traditional off-chip analytic assay methods [2] have granted them great acceptance by the research community. Typically, the application of manufacturing techniques for microelectronics in the implementation of micro analysis devices on silicon and glass substrates has favored their miniaturization and integration with sensors and actuators. Nevertheless, the reduction of production costs as well as the compliance to biosafety regulations to enable their use in clinical and environmental assays, have motivated the development of microfluidic biosensors of disposable materials [3]. Although the application of large scale production, low-cost materials such as paper or polymers has flourished in the field [4,5], the integration of the diverse capabilities necessary to promote the biorecognition reactions on a single microfluidic disposable substrate, still poses a challenge for every new biosensing application.

Polymer materials have been largely used to develop monolithic substrates for devices designed for biosensing, cell and tissue cultivation, separation and reaction [6,7]. These materials afford the implementation a variety of structures, while keeping desirable features such as low cost production, chemical resistance and mechanical strength [5,8]. Thermoplastic materials, in addition to these qualities, offer flexible manufacturing possibilities through different techniques (milling, hot embossing, injection molding) [9]. Although semi-crystalline and amorphous thermoplastics have been employed to produce molded parts, the later type of material displays a wider molding temperature range, which allows a more flexible usage of replication techniques [10]. Amorphous thermoplastics such as polystyrene (PS), polycarbonate(PC), polyvinyl chloride (PVC), poly(methyl methacrylate) (PMMA), cyclic olefin copolymer (COC) and cyclic olefin polymer (COP) are found as usual materials of microfluidic substrates [5,7,9].

Particularly, COC material has earned great acceptance due to their low water absorption, low auto fluorescence and high chemical resistance [11,12,13]. The material is produced from the co-polymerization of norbornene and ethylene monomers, whose proportion modulates the transition temperature and mechanical properties of the resulting copolymer [14,15,16] and produces different material grades, as available in the market. Its high transparency and low autofluorescece among the thermoplastics turn COC into one of the most popular substrate materials for production of fluorescence-based biosensors.

Pathogens are a persistent target in the biosensing research community due to the important awareness on their detection and control. The research on bacteria has largely employed strains of *E. coli* as model organism, not only due to its culturability and ubiquity, but also because some of its varieties are highly infectious and pathogenic, so their detection is reasonably well motivated. Detection of *E. coli* has been also a topic in the development of biosensors, on which several approaches can be found in the literature [17,18,19,20]. The detection of bacteria and other pathogens by means of nucleic acids in environmental assays [21] or in the detection of diseases biomarkers [22], has been discussed in recent years. Due to their high affinity, nucleic acids are regarded as an important type of biomarker for detection of biological targets [23]. Important efforts have been made to bring the analysis of bacteria by nucleic acids into microfluidic biosensors [24,25,26]. Nucleic acid biosensors profit from the selective binding between target nucleic acid pairs and complementary capture probes, resulting in remarkable specificity [27]. Nonetheless, while nucleic acid capture probes can be laid on the reactive surface of the biosensor at the fabrication stage, the retrieval of target nucleic acid during the assay requires preparation of the sample. In addition, detection schemes targeting bacteria RNA are very sensitive to rise of temperature due to RNA fast degradation. In spite of that, RNA detection provides a good indicator of the presence of recently active pathogens and therefore becoming used on environmental biosensors [21,28], motivating the close integration of retrieval, processing and detection of bacteria RNA in the same device.

Detection of the biorecognition events within a biosensor has been performed mainly by mechanical, electrical or optical means. Particularly, optical transduction by fluorescence detection has become a gold standard in the concerning research field [29,30,31,32]. Fluorescence detection is considered a versatile and sensitive method for detection and it is the most used detection method in micro total analysis devices (μTAS) [33]. In addition to detection, μTAS devices can perform sample preparation and conditioning functions, mostly integrated in a microfluidic chip. As example, lab-on-chip systems intended for nucleic acid amplification and analysis are capable to cary on on-chip sample purification, mixing of reagents, cell lysis and thermal cycling functions [34,35]. The integration of these capabilities in sinlge polymer substrate devices becomes challenging due to the control of off-chip flow actuatuors and precise monitoring and control of on-chip temperature [36,37]. Although substrates are made of low cost polymer materials, complete biosensors are typically fitted with metallic sputtered temperature sensors and actuators, which increases the manufacturing complexity and cost per unit of such devices. In order to reduce cost, the application of screen printed circuitry for temperature actuation and sensing can be explored. Currently, the usage of screen printed elements as electrodes for diverse biosensor application has been reported and discussed for electrochemical DNA biosensors [38,39].

The present work describes the design and prototyping of a disposable microfluidic biosensor. The device is fully made of COC and performs bacteria heat lysis for RNA retrieval, sample cooling and analysis reaction on the same substrate. Heat actuation and sensing is implemented by screen printed circuitry on the sealing foil and its effectiveness for heat lysis of cells is assessed. The device is tested as microfluidic biosensor of *E. coli* using fluorescence detection, with a total assay time of less than 1 h. The observed performance proves the potential application of a low-cost, disposable microfluidic biosensor at real-time, lab-on-chip based monitoring and control of bacteria pollution.

## 2. Materials and Methods

### 2.1. Biosensor Design and Preparation

**Figure 1:**
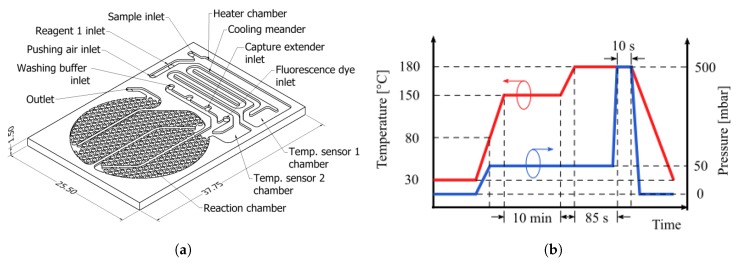
(**a**) Description of the manufactured biosensor. Dimension in milimeters. (**b**) Diagram of the applied hot embossing process parameters vs. time.

The microfludic biosensor was designed to host sample heating, cooling and detection reactions (total fluid volume 134 μL). In addition, it should allow temperature measurements on the running sample and reception of the reagents participating in the detection reaction, which are injected by off-chip pumps from reservoirs. The chip design concept is illustrated in Figure 1a. The surface on the reaction chamber (75 μL) has been patterned with obstacles in order to increase the surface-to-volume ratio and promote adsorption on the surface activated with reactive molecules [40]. The round shape of the reaction chamber was designed to fit under a 1″ diameter optical fitting. Partition walls in the reaction chamber aims to maximize the fluid sample transit time over the activated surface and by this way improve the occurrence of binding events and thus the biosensor effectivity. The channels, as well as the chambers, deepen to 300 μm. Before manufacturing rheological characterization of COC was conducted in order to estimate the material viscosity vs. temperature profile. From these results, the hot embossing process parameters were determined and applied as indicated in Figure 1b. COC plates, from TOPAS grade 5013 (transition temperature T${}_{g}$ of 142 ${}^{\circ }C$), were processed by hot embossing to structure the substrate.

### 2.2. Screen Printed Temperature Sensors

**Figure 2:**
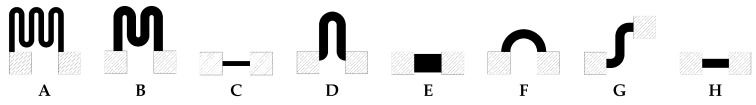
Carbon resistors test geometries. Carbon ink coverage on black. Dashed regions represent the overlap of carbon ink and silver ink for connection. Labels from A to H for further reference in Table 1.

Firstly, a characterization of screen printed material was conducted to determine their temperature sensitivity. Carbon ink (26-8203 Touchkey, SunChemical) was used to screen print temperature dependent resistive tests structures on COC foil, while silver ink (26-8204 Touchkey, SunChemical) was used to screen print electric contacts at the ends. Eight different types of geometry were printed on COC TOPAS 5013, 100 μm thickness foils, using mesh with 0.12 mm aperture (Sefar, Proell). The tested geometries are depicted in Figure 2, labeled with letters from A to H. Ten samples per geometry were manufactured and tested. The characterization was conducted on each sample by injecting 1 mA through the carbon resistor and measuring the resulting voltage drop with an analog input channel of a data acquisition board (NI USB-6361, National Instruments). The COC foil with the carbon resistor printed on it was laid over an aluminum plate on top of a hot plate, whose temperature was set to rise freely from room temperature up to 100 ${}^{\circ }C$. A second channel of the data acquisition board was connected to a PT-100 resistance temperature detector (RTD) to register the temperature on the aluminum plate. Resistance and temperature data were collected from each sample at each resistor type. Data from every sample were processed in MATLAB to obtain the polynomial fitting ($p_{1}x^{4}+p_{2}x^{3}+p_{3}x^{2}+p_{4}x+p_{5}$) and exponential fitting ($A\exp\left(Bx\right)+C\exp\left(Dx\right)$).

### 2.3. Cell Heat Lysis

The cell lysis efficiency was tested by flushing a solution of *E. coli* through the chip heater chamber, at different flow rates and under different heater powers. A sealed chip with a silver printed heater was prepared and its heater terminals connected to a current driver circuit. The heater driving signal consisted of a PWM signal (500 Hz, 5 V) produced by an analog output of the data acquisition board. The driving signal fed a NPN power transistor in common-emitter configuration to inject current in the heater. Measurements of current and voltage across the heater were taken using wired multimeters.

Every sample of *E. coli* was prepared from 100 μL of bacteria solution grown overnight in lysogeny broth (LB) at 34 ${}^{\circ }C$. The raw sample is then washed in 900 μL of PBS and bacteria pelletized by centrifugation (5000 revolutions per minute (RPM) during 4 min). A volume of 900 μL supernatant is replaced by the same volume of fresh PBS and the washing process is repeated twice more. After each sample was heat lysed, a 20 μL of acridine orange solution (0.7 mg/L in DI water) and 25 μL of propidium iodide (0.5 mg/L in DI water) are added to the lysed 100 μL volume of bacteria solution, and incubated at 37 ${}^{\circ }C$ for 20 min. After incubation the solution is washed in 900 μL of PBS, centrifuged at 5000 RPM for 4 min and removed of 900 μL supernatant, three times. At least three drops (0.5 μL each) from every processed sample were extracted and spotted on glass micro plates for microscope visualization. Filter sets for fluorescence imaging of acridine orange (480 nm/515 nm high-pass) and propidium iodide (494 nm/620 nm) were used in an epifluorescence microscope Nikon AZ-100. Images were taken with an attached camera Nikon D5100 (white balance fluorescence, exposure time 1 s, ISO 640). Recorded raw images were processed in ImageJ to count number of stained cells visible from each filter set at each sample. Counting was conducted by processing the 32-bit version of the raw images with a difference of Gaussian blurs filters and threshold selection. Resulting particles bigger than 3 pixels were counted.

### 2.4. Static Hybridization and Activated Biosensors

**Figure 3:**
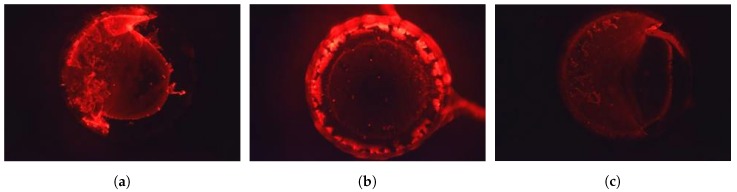
Fluorescence imaging from static hybridization tests. (**a**) Capture probe + extender molecule + fluorescence tag. (**b**) Off-chip RNA + fluorescence tag (no washed). (**c**) Capture probe + extender molecule + off-chip RNA + fluorescence tag (after washing).

Before closing the microfluidic structures, the reaction chamber surface was activated with capture molecules, using a customized technique based on the method described in [41]. As a manner of proof of concept of the hybridization over COC surfaces, tests on static hybridization were conducted. COC sample plates were hot embossed to produce 2 μL pits, which were bottom coated with capture probe molecules (custom oligomer purchased from Eurofins Genomics). Surface coated pits were spotted with a solution of extender molecule, dried in oven at 50 ${}^{\circ }C$ and washed. *E. coli* RNA was amplified and prepared to be spotted on the pits. In order to promote the hybridization, pits containing RNA were incubated, dried in oven at 50 ${}^{\circ }C$ and washed to remove unbound molecules. A solution with fluorescence molecules (DY-480 XL, 510 nm/648 nm) is added to tag binding places and reveal the hybridization function by fluorescence microscopy. Figure 3 exhibits the fluorescence imaging (filter set: 510 nm/650 nm).

### 2.5. Closing of Microfluidics

**Figure 4:**
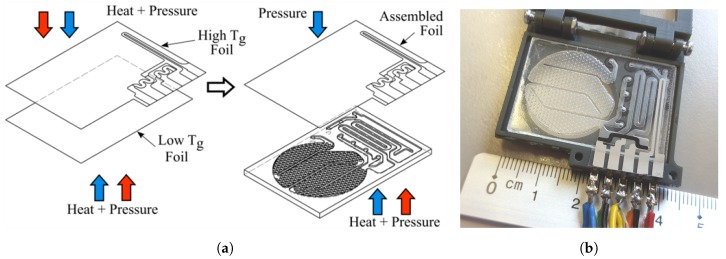
(**a**) Description of microfluidic closure: high temperature (red arrow), pressure (blue arrow). (**b**) Sample of a manufactured chip on a test holder.

Silver heater and carbon temperature sensitive resistors were screen-printed on a 100 μm thickness, T${}_{g}$ = 142 ${}^{\circ }C$, COC TOPAS 5013 grade foil, in the way the printed elements match the heater chamber and temperature sensor layouts. The printed side of the foil was hot pressed against a COC TOPAS 8007 foil T${}_{g}$ = 78 ${}^{\circ }C$, 500 mbar pressure and vacuum. Later the compound layer is aligned over the substrate layout and hot pressed on it (Figure 4a), by applying 100 ${}^{\circ }C$ from the bottom plate and 500 mbar pressure. A prepared biosensor is shown in Figure 4b, including a screen printed heater and temperature sensitive resistances.

### 2.6. Biosensor Experimental Setup

**Figure 5:**
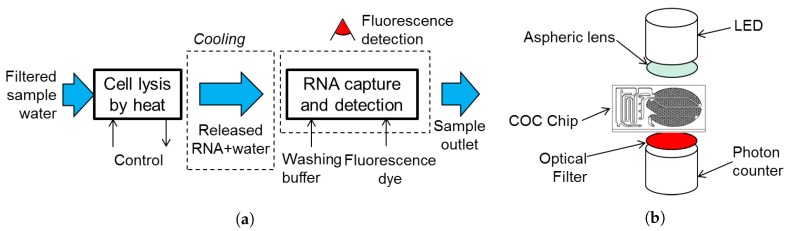
(**a**) Scheme of the experimental setup for testing the biosensor. (**b**) Scheme of the used fluorescence detection arrangement.

In order to test the biochip, a test bench was developed by research project partners. The test bench implemented the pumping functions, sample and reagents reservoirs, temperature control and fluorescence detection systems, as illustrated in Figure 5a. A sample volume of 10 L of water is taken at the test bench inlet, which is filtered from suspended particles and debris. After filtration, the bacteria content is pre-concentrated in 1 mL of sample, which is the volume to be injected in the biosensor. The test bench also controlled the heater and read the resistive temperature sensors signals. Moreover, it counts on a hot air blower to keep the chip at 50 ${}^{\circ }C$ during the test in order to temperate reaction chamber to a favorable temperature for RNA hybridization. An illustration of the optical system setup is presented in Figure 5b.

Before starting the biosensing experiments, several biochips were activated each with 25 μL of fluorescence tagged capture probe at different concentrations (0.01 pmol/mL, 0.1 pmol/mL, 1 pmol/mL and 10 pmol/mL) over the immobilized the reactive surface and closed with COC foils. Their fluorescence intensities were recorded using the optical detection system, in order to relate fluorescence intensity with density of capture probes.

Three types of biosensing tests were conducted using the chip in the test bench. First, a solution of *E. coli* in water was injected in the chip with the heater activated at the lysis power. Resulting lysed bacteria sample, containing released RNA, was collected at the chip output and analyzed in PCR, in order to assure the effectivity of the lysis method. Using a fresh activated chip, the second test consisted of the injection of a solution of water including off-chip prepared bacteria RNA. After injection of a solution including capture extenders, the off-chip RNA sample volume was flushed inside the chip, across the reactive surface in order to promote hybridization. Later, binding molecules tagged with DY-480XL fluorophore were injected to bind hybridized compounds. After washing of unbound molecules, the fluorescence intensity over the reaction chamber was measured by means of the optical detection system. The final type of test was conducted injecting a filtered water volume with pre-concentrated bacteria in it. On-chip heat lysis was activated to release the RNA from the injected bacteria. The lysed sample is flushed across the reaction chamber where the hybridization reactions take place. Injection of capture extenders, washing buffer and fluorophore followed the same sequence as described for the previous type. Fluorescence intensity is measured with a the same optical detection system.

## 3. Results

### 3.1. Screen Printed Temperature Sensors and Heater

**Figure 6:**
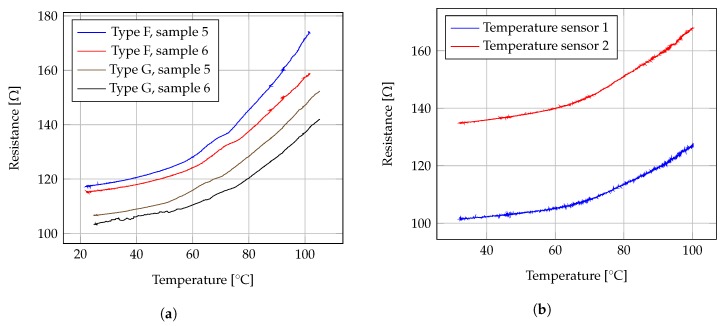
(**a**) Resistance vs. temperature profile from measured data at two test carbon printed resistors. (**b**) Measured data from samples of temperature sensors 1 and 2.

Figure 6a represents an example of the relation resistance vs. temperature drawn using measured data. Plotted data suggest an exponential or at least polynomial relation between resistance and temperature. In order to verify such indication, the root-mean-square error (RMSE) from each regression was taken as indicator of fitness. Results from geometries and regression statistics are summarized in Table 1.

**Figure 7:**
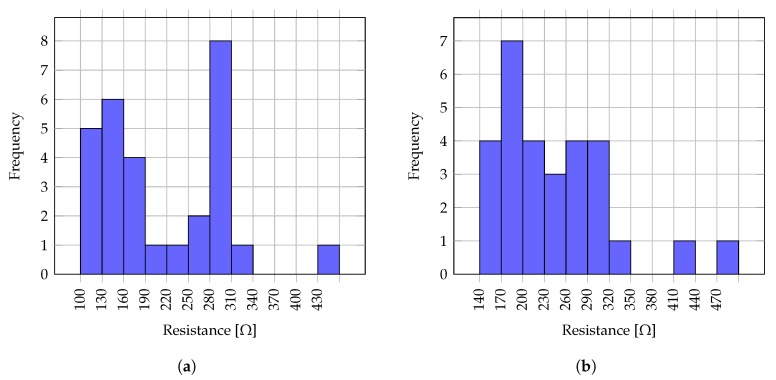
Histogram of resistances from 29 samples of carbon temperature sensors type 1 (**a**) and 2 (**b**).

**Figure 8:**
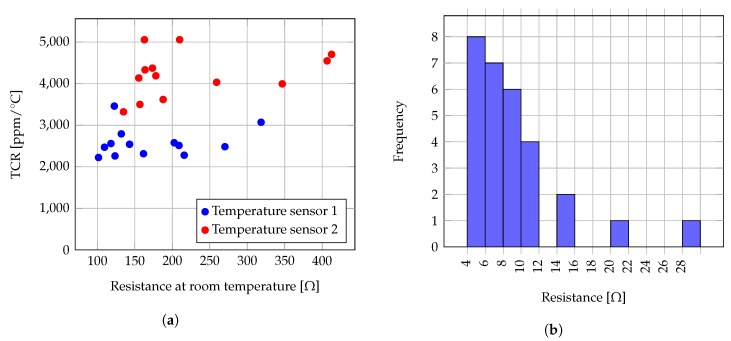
(**a**) Temperature coefficients of resistance (TCR) for samples of temperature sensor 1 (blue) and 2 (red). TCR calculated from room temperature to 95 ${}^{\circ }C$. (**b**) Histogram of resistances from 29 samples of silver ink printed heaters.

Temperature sensors and heaters were prepared using the same screen printing technique and materials as used for the test structures. For each sealing foil on each device, two carbon temperature sensors and one silver heater were screen printed, matching the respective chambers depicted in Figure 1a. Batches of screen printed temperature resistors, designed for the biosensor layout, were measured and their temperature response characterized. Figure 6b plots the resistance vs. temperature profile from data measured at sample temperature sensors type 1 and 2. Resistance reproducibility was examined on a batch of 29 samples, and the collected values are represented in a histogram for temperature sensors type 1 in Figure 7a, for temperature sensors type 2 in Figure 7b. A batch of 13 samples of COC foils with printed temperature sensors types 1 and 2 was characterized employing the same technique described for the test foils, and their temperature coefficients of resistance (TCR) were calculated between room temperatute and 95 ${}^{\circ }C$, obtaining the values registered in Figure 8a.

**Figure 9:**
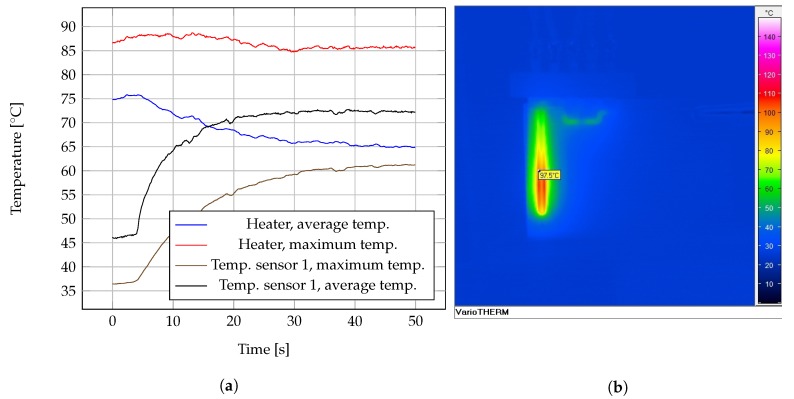
(**a**) Temperature time profile from infrarred imaging on working microfluidic device, 0.1 mL/min. (**b**) Thermal infrarred imaging displaying heater temperature emission at 240 mA.

A batch of silver screen printed heaters on COC foils were also measured. Values for a batch of 29 heaters are presented in the histogram in Figure 8b. The performance of printed heaters as heat source in a close chip was also tested. Using readings obtained from an thermal infrared camera (VarioTherm II, InfraTech GmbH), Figure 9a plots the temperature profile on time over the heater temperature sensor 1 chambers, given an inflow fresh water at time 0 s and 0.1 mL/min of flow rate. Figure 9b depicts one infrared imaging taken over the chip when heater was powered at 240 mA.

### 3.2. Cell Heat Lysis

**Figure 10:**
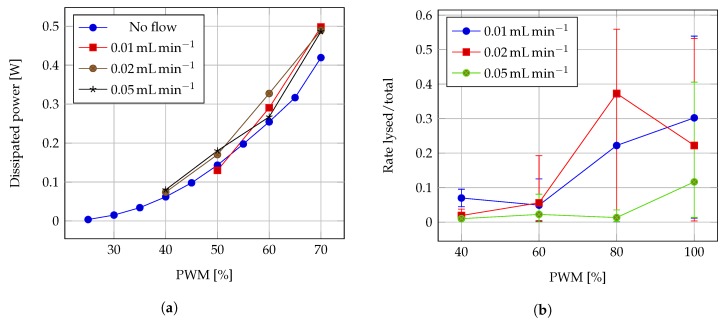
(**a**) Dissipated power on heater during lysis tests. (**b**) Cell lysis performance at different flow velocities.

**Figure 11:**
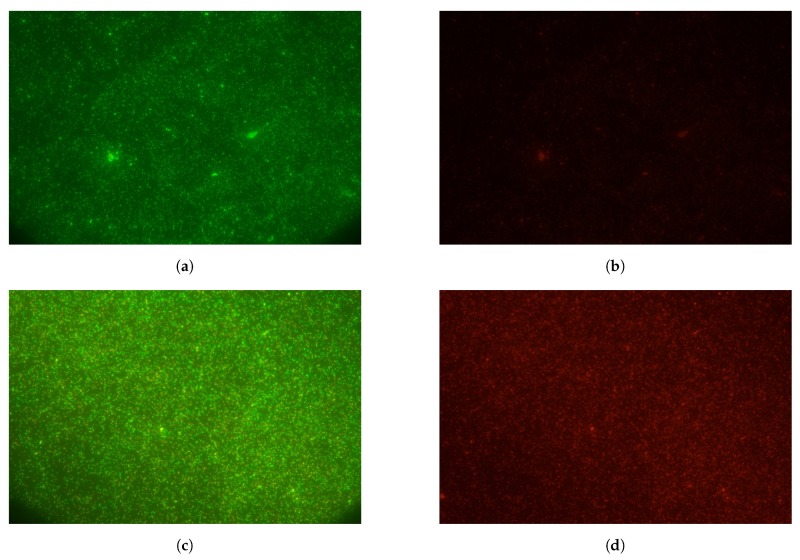
Fluorescence imaging on *E. coli* samples after heat lysis. Samples processed at 0.05 mL/min and heater powered at PWM 60%, acridine orage emission (**a**) and propidium iodide (**b**); and samples processed at 0.02 mL/min and heater powered at PWM 80%, acridine orage emission (**c**) and propidium iodide (**d**).

The dissipated powers at different excitation PWM duty cycles were calculated using the current and voltage readings on the heater (2.5 Ω) with no flow and during tests at 3 different flows, as plotted in Figure 10a. Following test preparation described in Section 2.3, the ratio of counted cells at propidium iodide imaging and acridine orange imaging from each test was calculated. Average rates and extreme rates for each test configuration are registered in Figure 10b. Examples of recorded fluorescence imaging are shown in Figure 11.

### 3.3. Fluorescence Tests on Biosensor

Preliminary fluorescence readings from fluorescence tagged capture probes are registered in Table 2. The presented results are average of the intensity value over the reading time (photon counter generated a reading every 100 ms). Test were conducted on an empty closed COC chip and four concentrations of capture probe solution. Measurement was taken at LED deactivated (background on dark), and at LED activated. The difference between the recorded intensity in presence of capture probe concentrations and empty COC chip, both at LED activated, gives a estimation of the net fluorescence increment due to presence of fluorescence dyed capture probes. Also it can help to correlate the detected intensity with amount of observed fluorophore.

Biosensing tests on the biochip produced fluorescence intensity readings, obtained with the test bench setup described in Section 2.6, and registered in Table 3. Background LED OFF measurements are those taken without LED activation. Given that the optical setup housed the chip in a dark chamber, these values give an appraisal of residual light and instrument noise. Background LED ON values represent the fluorescence intensity collected with LED activated, but no fluorophore molecule has been injected. Maximum intensities are assumed the ones taken at the moment of maximum fluorophore concentration in the reaction chamber. The last category reports the signal intensity after hybridization and washing, which reflects the fluorescence intensity of the bound remaining fluorescence tags, after unbound molecules have been washed off.

The three types of biosensing tests are encompassed in the results in Table 3. Capture Probe + Capture Extender corresponds to the test of hybridization between the capture probes on the chip surface and a fluorescence tagged capture extender. The second type consisted of the assessment of the hybridization of the Capture Probe + Capture Extender compound (without fluorophore) and off-chip amplified bacteria RNA (fluorescence tagged). Last type comprises fluorescence intensity readings taken during the complete assay sequence, capture probe hybridized with capture extender, on-chip heat lysed retrieved RNA and fluorescence tag.

## 4. Discussion and Conclusions

The COC rheological characterization results proved to be critical for the determination of the hot embossing parameters. Its results offer the potentiality to estimate process parameters for different geometries and feature sizes, however constrained by the mold manufacturing possibilities. On the other hand, the used foil sealing technique, consisting on hot pressing applying temperature from bottom substrate side, proved to obtain better resulting channel integrity in comparison to applying the molding heat from above the foil. Probable reason is the selective bonding heat distribution, since it comes from below the substrate to just the contact area between COC foil and COC substrate.

The implementation of screen printed structures aimed to complete the low-cost implementation of a thermoplastic biochip, which would make its disposability reasonable from an economical point of view. For characterization of tests structures, data from Table 1 show, as expected, a lower initial resistance for shorter lengths. The least variability is found towards the smallest L/W ratios. This finding suggests that probably the larger structures are more susceptible to printing process variations, which may lie on factors like the mesh development and processing, or the uniformity of squeegee force to evenly distribute the carbon paste over a larger area. Carbon printed test structures, as well as the temperature sensors for the biochip, showed a consistent exponential-like resistance vs. temperature profile and TCR ($\mu_{0}$ = 4677.55 ppm/${}^{\circ }C$ and $\sigma_{\mu }=264.53$ ppm/${}^{\circ }C$). Nevertheless, the room temperature resistance variance between different types of structures substantially fluctuated. Exponential and polynomial fitting were calculated as described in Section 2.2. Comparison of the obtained RMSE coefficients suggests that a fourth-degree polynomial regression fits better than an exponential regression for each of the analyzed types. The validation of the best fitting model sought for a mathematical model to predict the resistance variation against temperature. However, the determination of a set of polynomial coefficients that adapts to every case is still inconclusive. The best prediction extracted from measurements, is that the calculated resistance value at 95 ${}^{\circ }C$ is between 1.31 and 1.35 times the resistance at room temperature.

Silver screen printed heaters resistance, on the other hand, showed good repeatability ($\mu_{0}=$ 7.9 Ω, σ= 1.09 Ω from the results in Figure 8b). Figure 9a showed the heater can produced temperatures around 90 ${}^{\circ }C$ in water flowing at 0.1 mL/min. Tests conducted on printed heaters under no flow conditions showed dissipated powers up to 1.0 W and temperatures up to 175 ${}^{\circ }C$ for short time, demonstrating the screen printed device can still withstand demanding operation. It is reasonable to assume that the faster the fluid flow, the lower the average temperature attainable within the heater chamber, which may reduce the effectivity of the cell lysis, as it can be deduced from the results in Figure 10b. Values of 0.01, 0.02 and 0.05 mL/min were applied during the cell lysis test from Section 2.3. However, a higher flow rate was applied during the RNA hybridization experiments described in Section 2.6, due to the limitations to control the upstream pumps in the test bench at lower operation frequencies. This motivates a more comprehensive analysis of the effects of fluid flow in the temperature distribution, given the biochip geometry and materials, in a future work.

**Figure 12:**
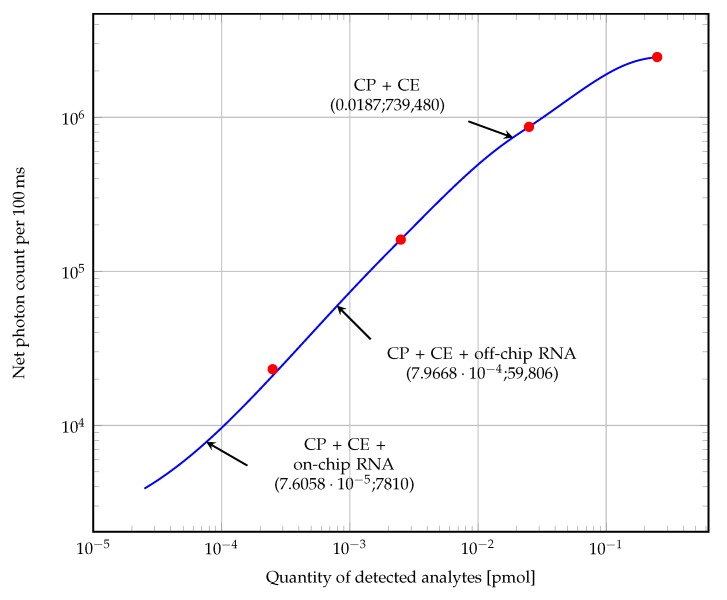
Calibration curve of detection, based on data from Table 2 (red circles). Measurement difference between LED ON and empty COC background (net fluorescence). Interpolation using MATLAB smoothing spline interpolation (blue line).

The preeliminary tests using the calibration samples obtained the fluorescence average intensities registered in Table 2. From the calibration net fluorescence intensities a calibration curve is built in Figure 12. The blue plot represents the interpolation of the net fluorescence intensities while the red dots are the average fluorescence intensity of calibration data. Estimation of the amount of captured analytes in mol is then regressed in the curve given the calibration volume of 25 μL and net fluorescence intensities, which are found by subtracting the detected intensity with the respective background value on LED ON. The interpolation curve was calculated using MATLAB fitting function model set as ‘Smooth spline’, with smoothing parameter p= 0.9999. The net results from experiments registered in Table 3 were regressed into the calibration curve and the respective amount of detected analyte estimated. Observed net intensities did not follow a linear relation, but incremental with negative concavity. For better visualization, data was plotted on logarithmic scales.

The estimation of the limit of detection (LoD) is formulated in the norm ISO-11843 as the determination of “*the lowest quantity of substance that can be distinguished from the absence of substance (blank value) within a stated confidence limit*” [42,43]. Since the fluorescence signal measurement is subject to error, the estimation of limits is treated with an statistical approach [44]. Be α the probability of *false positive* (type I error) and β the probability of *false negative* (type II error) in the determination of the detection limits. Assuming an univariate relation between fluorescence intensity and analyte concentration, a linear regression was estimated using measured data at the first three calibration concentrations (blank chip, 0.01 pmol/mL and 0.1 pmol/mL) of the form:

(1) $$\hat{y}=b_{0}+b_{1}x+\epsilon$$

where $\hat{y}$ is the calculated fluorescence intensity, $b_{0}$ and $b_{1}$ are the intercept and slope of the linear regression respectively, and ϵ is a random error signal with null mean and normal distribution. Taking α= 0.05 (probability of detection of analyte when there is none), the *decision limit* is calculated as [45]:

(2) $$y_{c}=b_{0}+w_{0}S_{y|x}t_{\alpha ,N-2}$$

where $w_{0}S_{y|x}$ is the standard deviation of $b_{0}$ and $t_{\alpha ,N-2}$ the one-tailed value of a t-Student’s distribution with N−2 degrees of freedom for α. The calculation of Equation (2) results in $y_{c}=$ 188,256, which is regressed as $375.733\times 10^{-6}$ pmol/mL. The *capability of detection* or *detection limit* on the other hand, is the minimum value of analyte on which the risk of non-detecting an analyte, when it truly present, is β. Assuming β= 0.05, the respective fluorescence value is $y_{d}=$ 188,858, and the LoD becomes $757.86\times 10^{-6}$ pmol/mL. Given a calibration sample volume of 25 μL, the amount of analytes corresponding the decision limit and LoD are $9.393\times 10^{-6}$ pmol and $1.895\times 10^{-5}$ pmol respectively. The theoretical LoD calculated for this device in terms of concentration can be expressed as 757.86 fM. This performance is compared with some references from the literature as shown in Table 4. Still more tests over controlled concentrations of bacteria have to conducted to obtain an accurate estimation of performance parameters in CFU. These results will allow better comparison with relevant works in the literature, where the reporting of concentrations in CFU per mL is standard for bacteria biosensors.

Nevertheless, from the limited information about the bacteria concentration still some observations can be drawn. The estimated amount of detected analytes, at the off-chip RNA assay, obtained a net photon count around 10.5 times greater than from the on-chip RNA assay. Taking into account that at the former assay the introduced concentration of bacteria was 50 times larger than at the on-chip RNA test, it can be deduced that there are several factors yet to be considered. Probably, the effect of the lysis temperature and injection sample flow played a detrimental role in the completion of hybridization, whose real impact might be better explained by further experiments. From the analytical point of view, interpolation model leaves room for loose predictions on beyond the extreme points, motivating the collection of more data towards lower concentrations for future works. From the instrumental point of view, the effect of variables such as the sample injection flow rate and the observed formation of foam in the hybridization buffer, probably impacted negatively the hybridization reaction efficiency. The study and control of this variables were out of the scope of this work, and its conduction on the designed device is a motivation for future works.

As proof of concept, this work showed that a RNA based bacteria detection concept can be implemented on a single substrate, disposable material, microfluidic chip, suggesting its potential for further customization and optimization for detection of another pathogenic bacteria species. 

## Figures and Tables

**Table 1:** Statistics from characterization of carbon resistors represented in Figure 2.

Type	Length [mm]	Width [mm]	Ratio L/W	Resistance at Room temp.		TCR (from Room temp. to 95 ${}^{\circ }$C)		RMSE Polynomial Fitting		RMSE Exponential Fitting	
				$\mu_{0}$ [Ω]	σ [Ω]	$\mu_{0}$ [ppm/${}^{\circ }$C]	σ [ppm/${}^{\circ }$C]	$\mu_{0}$	σ	$\mu_{0}$	σ
A	28.14	0.6	46.9	997.59	115.86	4832.48	1851.4	2.615	1.292	3.527	1.810
B	18.57	1.0	18.57	286.74	40.24	4354.41	436.25	0.7923	0.409	1.099	0.411
C	3.0	0.5	6.0	138.88	15.76	5118.88	691.73	0.315	0.0624	0.604	0.162
D	10.71	1.0	10.71	166.06	19.86	4534.84	531.51	0.438	0.110	0.574	0.184
E	3.0	2.0	1.5	32.88	3.73	4436.20	509.59	0.148	0.101	0.183	0.111
F	6.28	1.0	6.28	121.22	7.02	4863.82	450.97	0.337	0.0853	0.483	0.133
G	6.14	1.0	6.14	114.86	7.37	4393.73	437.12	0.312	0.0735	0.511	0.176
H	3.0	1.0	3.0	65.18	5.30	4885.04	441.92	0.304	0.386	0.4278	0.3799

TCR: Temperature Coefficient of Resistance. RMSE: Root-Mean-Square Error.

**Table 2:** Average fluorescence intensities, in photon counting per 100 ms, at different concentrations of surface fluorescence tagged capture probes. Sets of 49 measurements each concentration.

	Empty COC Chip	0.01 pmol/mL	0.1 pmol/mL	1.0 pmol/mL	10 pmol/mL
Background LED OFF	54,567	62,784	64,853	61,896	57,863
Signal at LED ON	184,160	207,330	344,960	1,051,400	2,646,400
Difference (LED ON minus LED ON, empty COC)	-	23,170	160,800	867,240	2,462,240

**Table 3:** Average fluorescence intensities in photon counts per 100 ms.

	Capture Probe + Capture Extender	CP + CE + off-Chip RNA (1 × 10${}^{10}$ CFU/mL)	CP + CE + on-Chip RNA (2 × 10${}^{8}$ CFU/mL)
Background LED ON/LED OFF	249,550/35,917	241,840/34,966	232,190/41,635
Maximum intensity with detection solution (before washing)/LED OFF	1,074,800/44,278	2,138,809/32,303	1,300,000
Signal after hybridization and washing/LED OFF	989,030/44,894	301,646/41,685	240,000

**Table 4:** Performance comparison from selected references of fluorescence biosensors.

Target	Detection	Limit of Detection	Year, Reference
*Escherichia coli* and *Escherichia faecalis*	PCR + surface hybridization	50 pM	2002, [34]
Dengue Virus	Magnetic bead-based sandwich	0.125 n M	2005, [46]
DNA	Ag surface DNA hairpin probes.	500 pM	2012, [47]
*E. coli*	Immunoassay bead-free detection with quantum dots	10 CFU/g	2012, [48]
*Legionella* spp.	Hybridization on magnetic beads	1.8 a M	2015, [49]
*E. coli*	Immunoassay on magnetic nanoparticles	30 CFU/mL	2016, [50]
*E. coli*	RNA Hybridization on surface by capture probes	757.86 f M	**This work**
